# Alteration of exosomes secreted from renal tubular epithelial cells exposed to high-concentration oxalate

**DOI:** 10.18632/oncotarget.21517

**Published:** 2017-10-04

**Authors:** Ziqi He, Xiaofeng Guan, Yunlong Liu, Zhiwei Tao, Quan Liu, Jihua Wu, Yaoliang Deng

**Affiliations:** ^1^ Department of Urology, First Affiliated Hospital of Guangxi Medical University, Guangxi, P. R. China

**Keywords:** exosomes, altered secretion, kidney stone, high-concentration oxalate, renal tubular epithelial cells

## Abstract

Oxalate (Ox) is a metabolic end product that is produced by the kidneys and is associated with several pathological conditions. The accumulation of oxalate in the body is one of the factors that lead to calcium oxalate kidney stones. To simulate the high-concentration Ox environment *in vivo*, we established an *in vitro* model of high Ox using renal tubular epithelial (HK-2) cells. Cell viability and proliferation were assessed to evaluate the effects of various concentrations (0, 0.25, 0.5, 1, 2, 4, 5, 8 and 10Mm) of Ox on HK-2 cells to select the optimum concentration and time to extract the exosomes. Treatment with 0, 1, or 2 mM Ox altered the morphology and secretion capacity of exosomes. As the concentration of Ox increased, peak and mean particle size decreased, but exosomes particle concentration, exosome RNA, and exosome protein increased. Size, distribution, and rate of secretion, as well as RNA and protein content, differed among extracellular vesicle subtypes. Furthermore, the three subtypes of exosomes delivered different signal factors in the microenvironment. We therefore speculated that three subtypes of exosomes may play differing roles in intercellular signal communication and the formation of CaOx kidney stones.

## INTRODUCTION

Kidney stone is a serious disease which is harmful to human health worldwide [1]. Pathogenesis of the disease remains unclear. Oxalate (Ox) is known to affect renal tubular epithelial (HK-2) cell growth. Several studies have suggested that Ox and CaOx crystals interact with kidney epithelial cells, leading to HK-2 cell injury, triggering cascade reaction, and, ultimately, stone formation [2, 3]. As an important mediator of intercellular signal communication, exosomes play a vital role in many diseases [4–6]. We hypothesized that exosomes might also play a critical role in stone formation. Exosomes are microvesicles widely found in body fluids that act as carriers for the transfer of DNA, mRNA, non-coding RNA, and lipid carriers. Exosomes also regulate the physiological function of target cells [7]. The secretion of exosomes is closely related to cell viability; exosomes have also been found in the urine of patients with CaOx kidney stones. The presence of exosomes in the urine seems to reflect underlying cellular processes that may be associated with stone risk [8]. Exosomes extracted from the urine are susceptible to changes in cellular origin, temperature and pH [8–11], rendering experimental results prone to variation. We sought to optimize the extraction and identification of exosomes using HK-2 cells exposed to high concentrations of Ox. We found that exosome size and secretion varied under different concentrations of oxalate [12], suggesting that three groups of exosomes may carry different signaling molecules and may play different roles in the micro-environment. This discovery may allow us to explore the mechanisms of signal transduction and targeted regulation of exosomes in stone formation.

## RESULTS

### High concentrations of Ox decrease cell vitality

**Figure 1:**
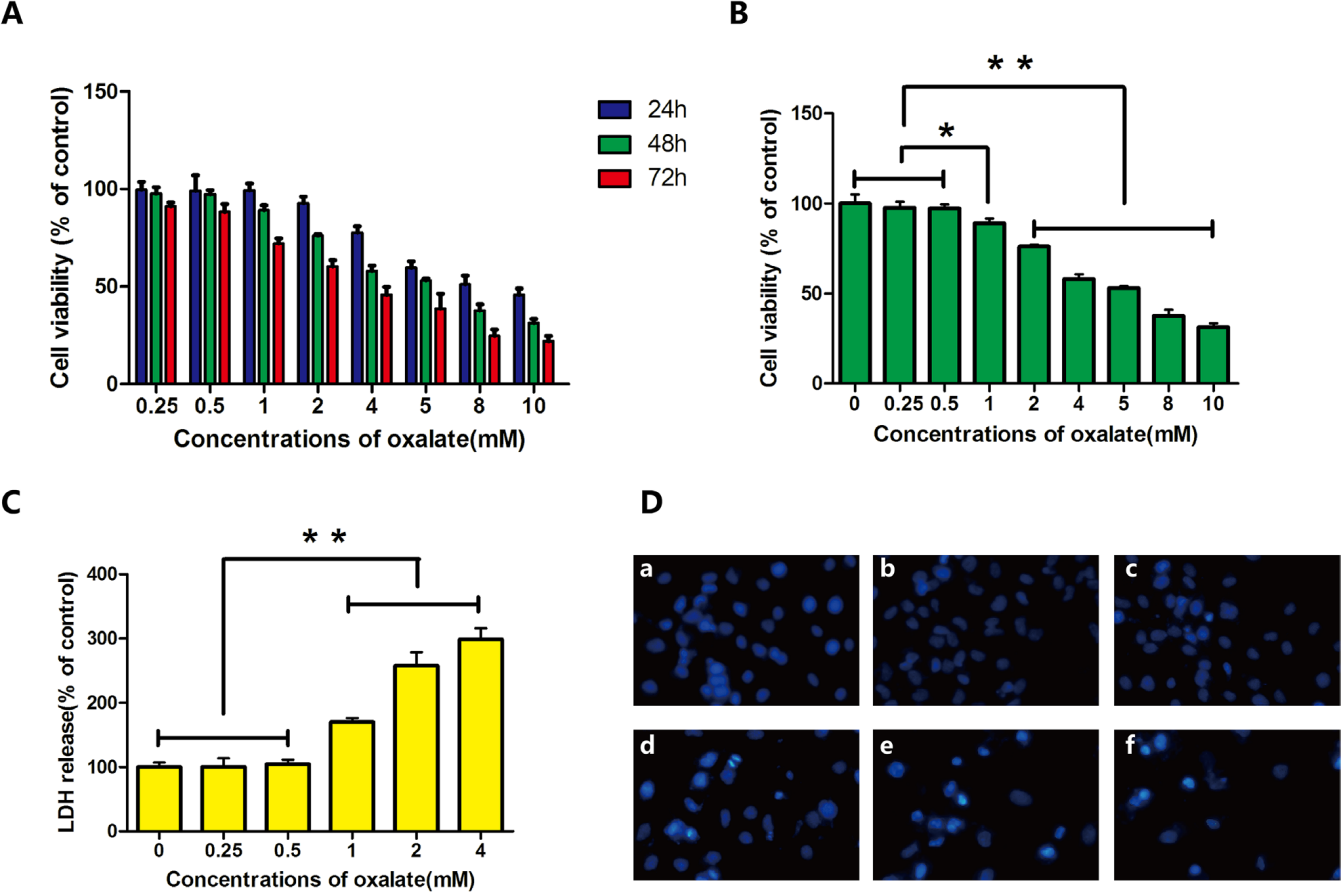
Cytotoxicity and cell damage caused by Ox **(A)** HK-2 cells were exposed to different concentrations of Ox, for various durations of exposure, to assess effects on cell viability. **(B)** HK-2 cells were exposed to different concentrations (0, 0.25, 0.5, 1, 2, 4, 5, 8, and 10 mM) of Ox for 48 h. Cell viability was determined by CCK-8 assay. **(C)** Six concentrations (0, 0.25, 0.5, 1, 2, and 4 mM) were used to study LDH release. **(D)** Cells in culture were examined by fluorescence microscopy following staining with DAPI. (a) Cells treated with 0 mM Ox for 48 h. (b, c) Cells treated with 0.25 or 0.5 mM Ox were similar to cells exposed to 0 mM Ox. (d, e) Cells treated with 1 or 2 mM Ox showed condensed chromatin. (f) Cells treated with 4 mM Ox showed condensed chromatin. Most showed signs of cell death (magnification, ×100). Bar: SD, ^*^, *P*<0.05 and ^**^, *P*<0.01 (B, C).

In this study, cells were exposed to different concentrations of Ox (0, 0.25, 0.5, 1, 2, 4, 5, 8, or 10 mM) for 24, 48, or 72 h. Cell proliferation and cytotoxicity were evaluated with the Cell Counting Kit-8 (CCK-8) assay, which exhibits high sensitivity and involves no radioactivity [13]. Cell damage and cytotoxicity varied among groups but displayed similar trends over time. Comparison among groups revealed 48 h as the optimal duration of treatment (Figure 1A). Ox concentrations ≥1 mM damaged HK-2 cells, in a concentration-dependent fashion (Figure 1B).

### High-concentration Ox increases lactate dehydrogenase

Lactate dehydrogenase (LDH) is a classic marker of cell damage and cytotoxicity. Results of the CCK-8 assay showed that Ox concentrations ≥4 mM obviously decreased cell viability. Subsequent experiments were therefore performed with 0, 0.25, 0.5, 1, 2, or 4 mM Ox. The release of LDH increased after exposure to ≥1 mM Ox; this effect was dose dependent (Figure 1C).

### High-concentration Ox increases apoptosis

**Figure 2:**
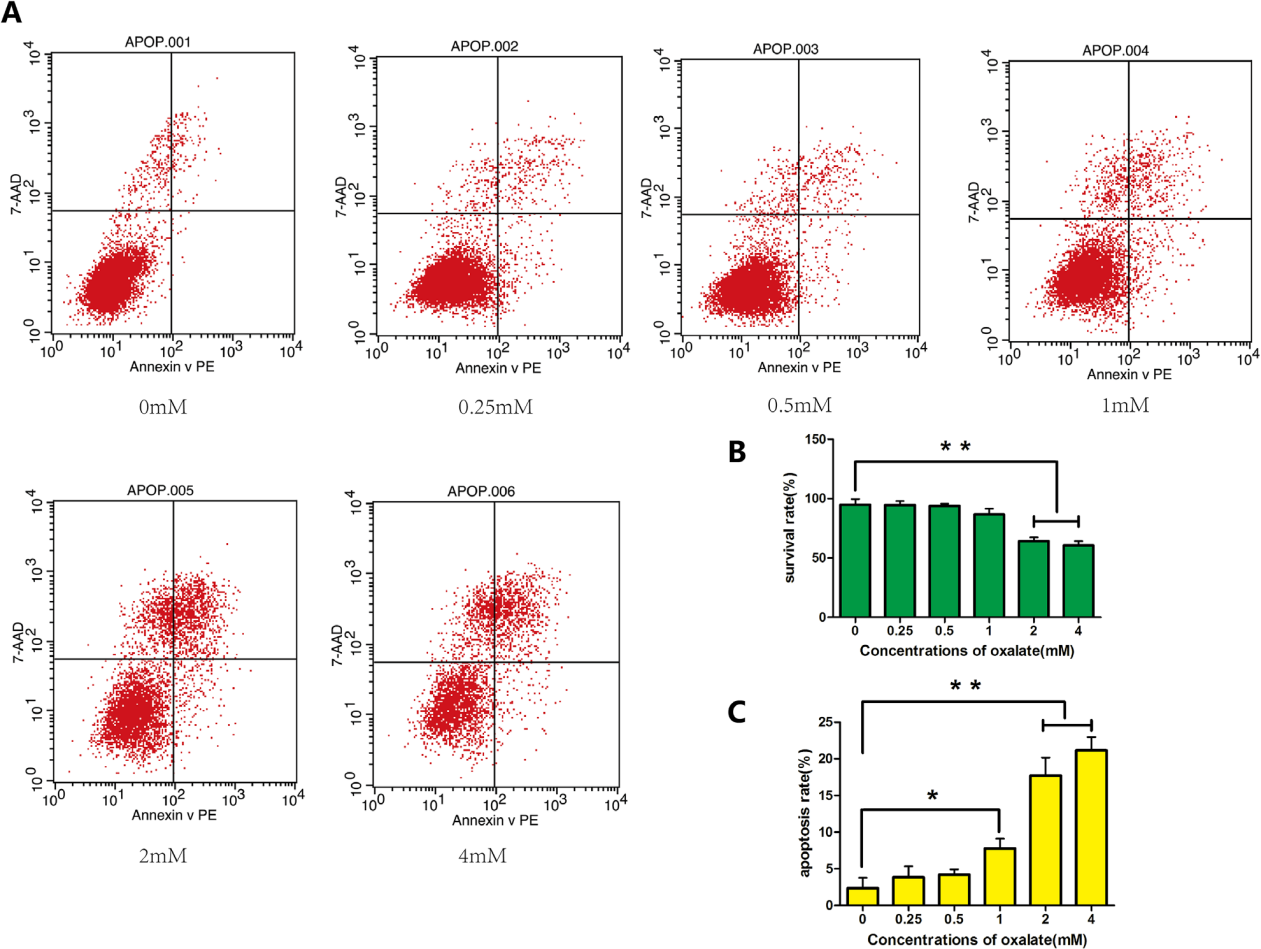
Apoptosis of HK-2 cells as detected by flow cytometry **(A)** Apoptosis in cells treated with different concentrations (0, 0.25, 0.5, 1, 2, or 4 mM) of Ox for 48 h, as determined with flow cytometry. **(B)** Rate of cell survival decreased significantly in ≥2 mM Ox. **(C)** Rate of cell apoptosis increased in ≥1 mM Ox. Bar: SD, ^*^, *P*<0.05 and ^**^, *P*<0.01 (B, C).

Cells were exposed to varying concentrations (0, 0.25, 0.5, 1, 2, or 4 mM) of Ox, which induced cytotoxicity and cell apoptosis in HK-2 cells. After 48 h of treatment, cells exposed to 1, 2 or 4 mM had condensed chromatin (Figure 1D). The results of flow cytometry showed increased apoptosis in cells exposed to ≥1 mM Ox (Figure 2). DAPI staining revealed prominent necrosis in cells exposed to 4 mM Ox. Subsequent experiments were therefore performed using concentrations of 0, 1, or 2 mM Ox.

### Electron microscopy

**Figure 3:**
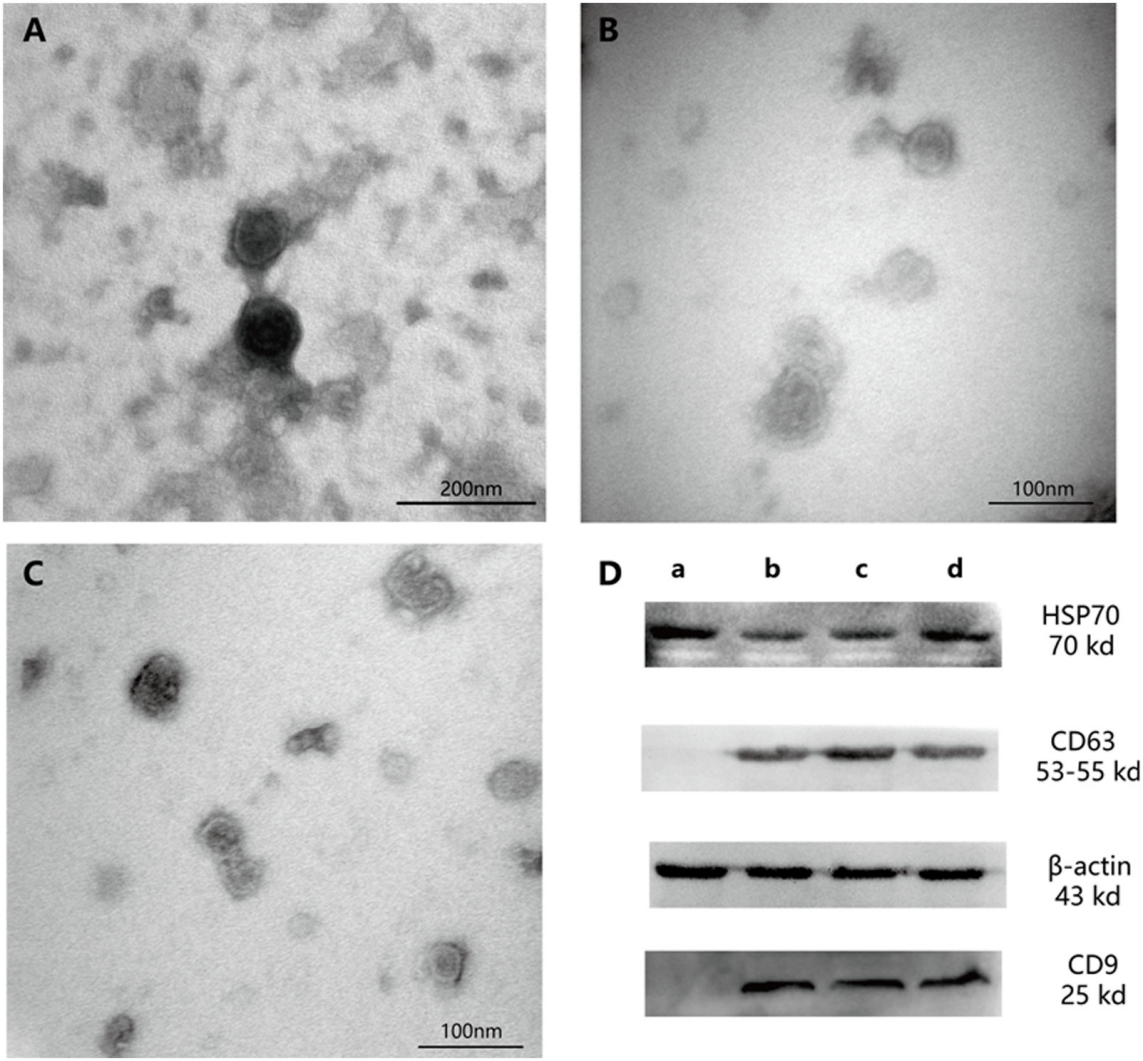
Exosomes as observed by electron microscopy and western blotting analysis **(A)** Exosomes exposed to 0-mM Ox treatment (magnification, ×200000). **(B)** Exosome exposed to 1-mM Ox treatment (magnification, ×300000). **(C)** Exosome exposed to 2-mM Ox treatment (magnification, ×300000). **(D)** Western blot analysis with exosome markers. (a) HK-2 cell sample. (b) Exosome exposed to 0-mM Ox treatment. (c) Exosome exposed to 1-mM Ox treatment. (d) Exosome exposed to 2-mM Ox treatment. Densitometry values for each protein were normalized to those of β-actin.

Electron microscopy can be used to visualize exosomes, which display cup-shaped morphology and a double-membrane structure (Figure 3A-3C).

### Expression of characteristic exosome proteins

Western blotting analysis has revealed that exosomes express significant levels of HSP70, CD63, and CD9 [14]. HK-2 cells (after produce exosomes) in this study expressed HSP70 but not CD63 or CD9 (Figure 3D).

### Effects of Ox concentration on size distribution and concentration of exosomes

**Figure 4:**
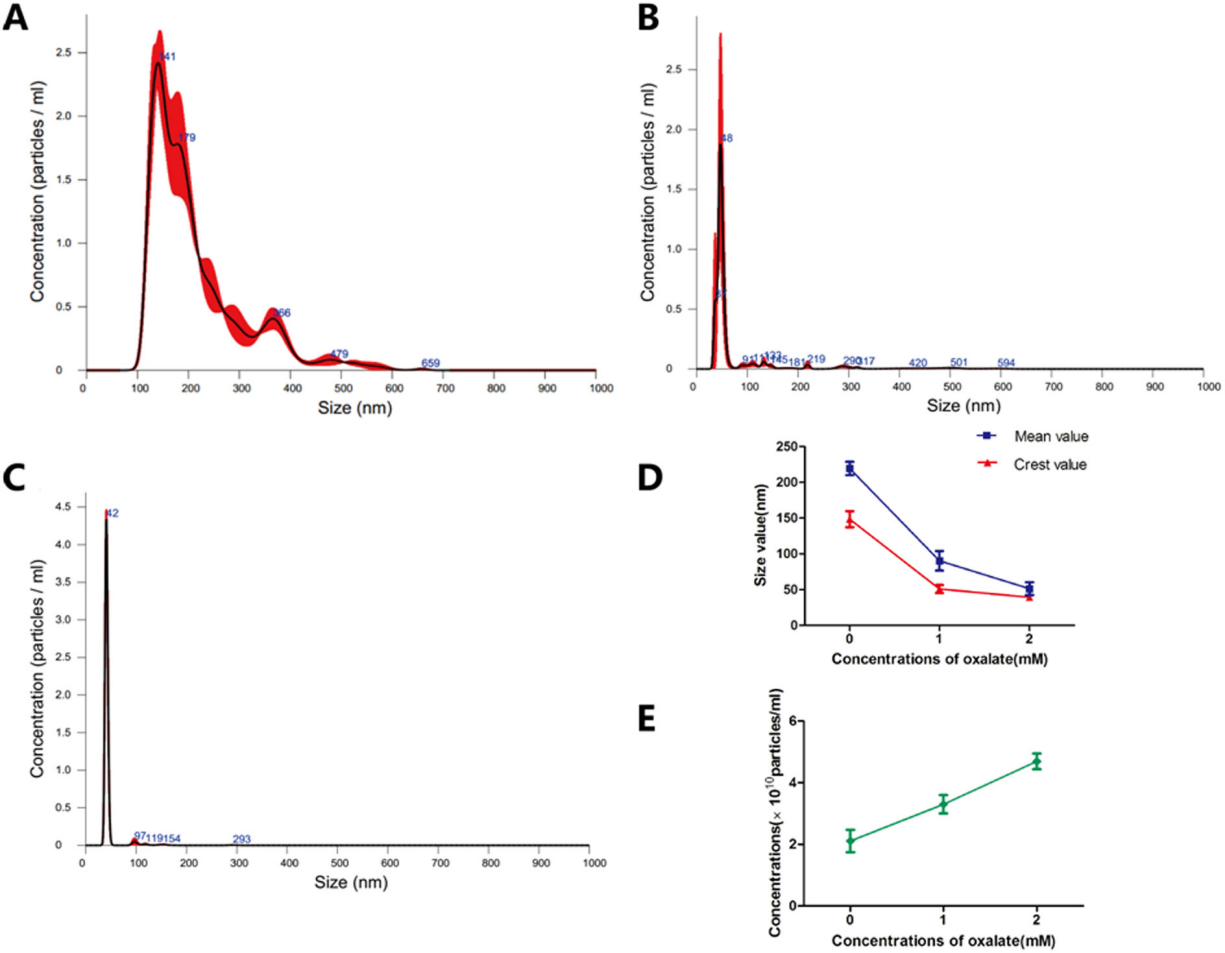
Exosomes were dynamically observed by nanoparticle tracking analysis **(A)** Exosome exposed to 0-mM Ox treatment. **(B)** Exosome exposed to 1-mM Ox treatment. **(C)** Exosome exposed to 2-mM Ox treatment. **(D)** Size distribution of three groups of exosomes. **(E)** Particle concentration for three groups of exosomes.

NanoSight NTA (Malvern Instruments Ltd, Malvern, UK) technology allows researchers to directly observe the dynamics of nanostructured particles in a state of Brownian motion. The Stokes-Einstein equation is used to determine particle size and concentration (particles/mL). This microscopic sample system provides accurate information about nanoparticles, including particle size, quantity, type and dispersion. The results showed that increasing concentrations of Ox decreased peak and mean exosome size and increased exosome concentration (Figure 4D-4E). In the 0-mM group, particle concentration was 2.11±0.36×10^10^particles/mL; peak size was (148.35±11.24) nm; mean size was (219.30±9.33) nm. In the 1-mM group, particle concentration was 3.30±0.29×10^10^ particles/mL; peak size was 50.85±5.44 nm; mean size was 90.35±13.79 nm. In the 2-mM group, concentration was 4.69±0.25×10^10^particles/mL; peak size was 39.35±2.33 nm; mean size was 51.30±8.91 nm (Figure 4A-4C).

### Effects of Ox concentration on exosome protein and RNA

**Figure 5:**
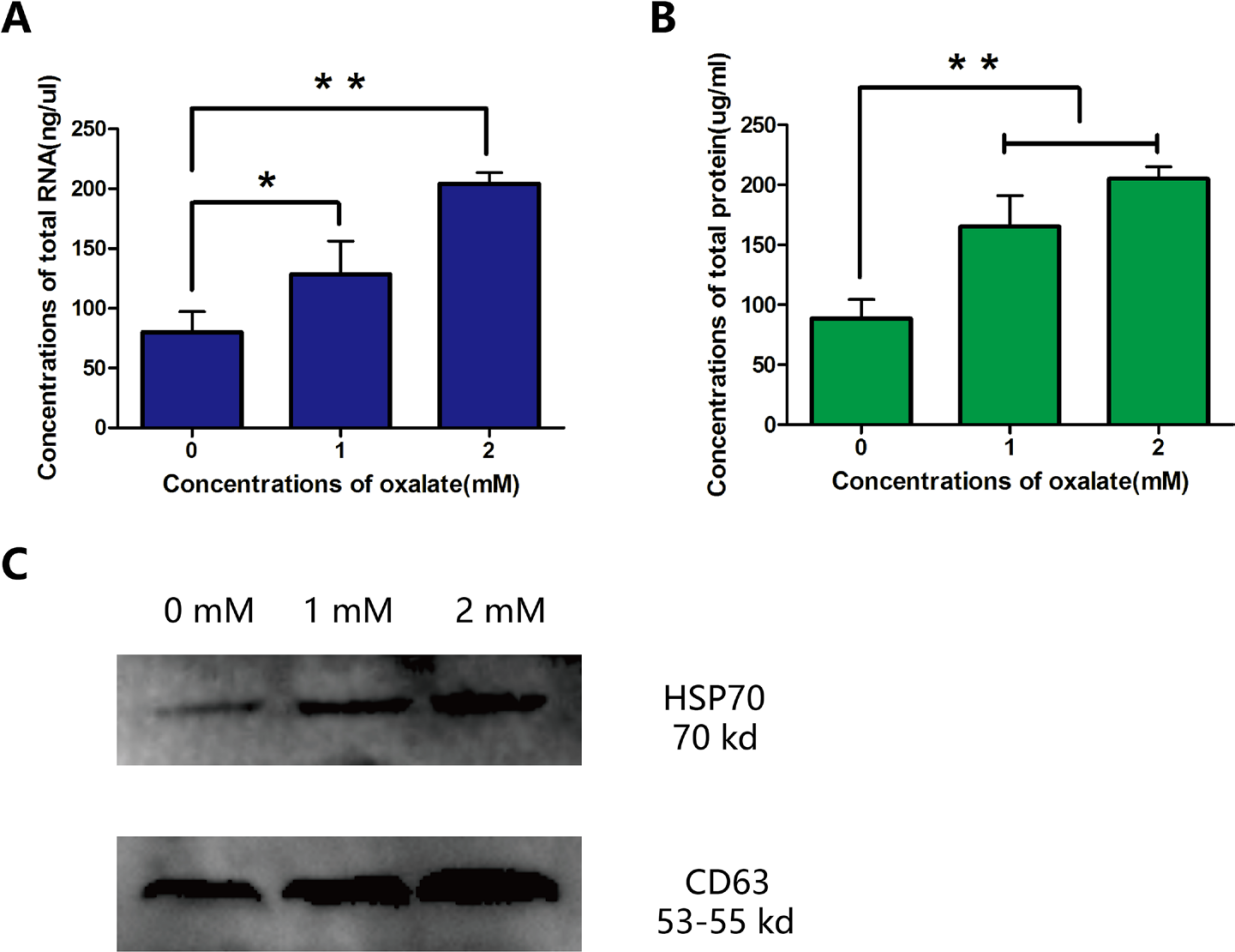
Quantification of RNA and protein in three groups of exosomes **(A)** Concentration of total RNA. **(B, C)** Concentration of total protein, as determined with BCA kit and western blotting analysis. Bar: SD, ^*^, *P*<0.05 and ^**^, *P*<0.01 (B, C).

Because exosomes are rich in protein and non-coding RNA, the quantification of RNA and protein can be used as an index for the secretion of exosomes. Original samples were lysed, and protein obtained (15 μl/sample) was subjected to western blotting analysis. RNA and protein levels of CD63 and HSP70 increased with Ox concentration (Figure 5).

## DISCUSSION

HK-2 cells exhibit oxidative stress injury following exposure to high concentrations of Ox. Studies have shown that this damage affects miRNA in HK-2 cells and activates certain pathways involved in the formation of CaOx kidney stones [15, 16]. Subsequent changes in the expression of miRNA may affect the secretion of exosomes [17]. For example, exosomes secreted into the urine of patients with type 2 diabetic nephropathy show upregulated expression of miR-15b, miR-34a, and miR-636. The specificity of these markers for type 2 diabetic nephropathy is as high as 100% [18]. Therefore, we have reason to speculate that Ox-damaged HK-2 cells may also secrete exosomes carrying specific miRNAs. To investigate this hypothesis, we sought to establish an *in vitro* model and therefore sought to determine the appropriate concentration of Ox and duration of treatment. Prolonged exposure to Ox or exposure to too high a concentration of Ox could induce degeneration and necrosis in HK-2 cells. Conversely, exposure to Ox that was too brief or too low in concentration could result in insufficient exosome alteration.

Isolation of the exosomes was a critical factor in this study. Recent advances in technology have provided improved techniques for isolation, including ultracentrifugation-based isolation techniques, size-based isolation techniques, immune affinity capture-based techniques, microfluidics-based isolation techniques, and exosome precipitation [19]. Ultracentrifugation-based isolation was selected based on exosome morphology, particle size distribution, and concentration. Although the steps were relatively cumbersome, the necessary equipment was available, and the procedure was amenable to use of a variety of cell culture media. Recovery rate was high, purity was moderate, and biological activity of exosomes was not influenced, which was especially beneficial for subsequent proteomic studies [20, 21]. A protocol for ultracentrifugation-based isolation reported previously was used, with modifications [22].

The results presented here show that Ox concentration affects the size and secretion of exosomes. These findings suggest that three subtypes of exosomes may carry different signaling molecules, playing different roles in a given microenvironment [23]. Exosomes are secreted through fusion with the membrane and secreted into the extracellular environment by exocytosis. These endogenous nanocarriers are 30-120 nm in diameter. Exosomes carry bioactive molecules such as RNA and proteins, which exist stably in the extracellular environment and regulate the metabolic pathways of target cells [24, 25]. Our results showed that higher concentrations of Ox damage HK-2 cells, inducing donor cells to secrete numerous exosomes. We began to consider whether exosomes act as a “messenger” or “trash bin” in environments with a high concentration of Ox [26]. We plan to conduct further research to elucidate these issues.

Exosomes have attracted increasing attention as an important mediator of intercellular signal communication. Various subtypes of exosomes appear to mediate different functions [12, 23, 27]. In this study, we established a protocol for extraction of exosomes secreted by HK-2 cells exposed to high-concentration Ox. We identified appropriate reaction conditions and isolation methods to facilitate further study of the relationship between exosomes and kidney stones. Our experiments revealed that exosomes secreted by HK-2 cells in various environments differed greatly in terms of shape, size distribution, and rate of secretion. Previous studies have reported that more limited particle size distributions are correlated with faster uptake by target cells and greater potency in the microenvironment [28]. Thus, we can assume that varying concentrations of exosome subtypes trigger different signaling molecules. For example, cells exposed to high-concentration oxalate (1 and 2 mM) secreted many exosomes of similar size, which facilitates absorption by target cells.

In conclusion, the exosomes secreted by HK-2 cells exposed to high-concentration Ox were extracted for the first time, using a novel protocol to differentiate three subtypes of exosomes. While exosomes are generally known to mediate intercellular communication, they may also function as microvesicles, removing signaling molecules that inhibit cell growth [26]. We have reason to believe that they will play a different role in the formation of CaOx kidney stones, and open up new horizons for studying the mechanism of CaOx stone formation.

## MATERIALS AND METHODS

### Cell culture

Human kidney epithelial (HK-2) cells were purchased from the Cell Bank of the Chinese Academy of Sciences (Shanghai, China) and cultured in DMEM/F12 (Gibco, Grand Island, Nebraska) medium supplemented with 10% fetal bovine serum (Lonsa Science SRL, Uruguay Origin, Rio, Brazil) and 1% antibiotics (Hyclone, Logan, Utah). Before Ox treatment, cells were maintained with free-serum for 6 hr.

### Cell exposure

Before treatment, Ox (Sigma, Saint Louis, Missouri) was resuspended in solution (Ox with free-serum DMEM/F12 medium) to eliminate aggregation between crystals. When 80% confluence was reached, cells were treated with different concentrations of Ox. For the CCK-8 essay, the following concentrations of Ox were used: 0, 0.25, 0.5, 1, 2, 4, 5, 8, and 10 mM). For LDH, DAPI, and apoptosis assays, Ox was administered at concentrations of 0, 0.25, 0.5, 1, 2, and 4 mM.

### CCK-8 assay

HK-2 cells were grown in a 96-well plate. Upon reaching 80% confluence, cells were exposed to different concentrations of Ox (100 μl/well) for 24, 48, or 72 h. Ten microliters of CCK-8 solution (Dojindo, Kamimashiki-gun, Kumamoto, Japan) was added to each well; plates were then incubated (37°C, 5% CO_2_) for 2.5 h. Absorbance was measured at 450 nm using a microplate reader (ThermoFisher, Vantaa, Uusimaa, Finland).

### LDH assay

LDH is a stable enzyme released to the blood when tissue is damaged or red blood cell hemolysis occurs. Because LDH is very stable, it is also widely used to assess damage and toxicity to cells. HK-2 cells were grown in 6-well plates. Upon reaching 80% confluence, cells were treated with different concentrations of Ox (2.5 mL/well) for 48 h; then, media was collected to measure levels of LDH. We used the lactate dehydrogenase activity assay kit (Sigma, Mannheim, Baden-Württemberg, Germany), in which LDH deacidifies NAD to NADH. Resulting NADH is specifically detected by colorimetry (with absorbance measured at 450 nm).

### DAPI staining

HK-2 cells were cultured in 6-well plates. Upon reaching 80% confluence, cells were exposed to various concentrations of Ox for 48 h. Cells were washed with PBS (Solarbio, Beijing, China) twice, then fixed with 4% paraformaldehyde (Solarbio, Beijing, China) for 15 min at room temperature, washed again with PBS (twice), stained with DAPI (Sigma, Saint Louis, MO) for 15 min, then washed once more with PBS (for 3 min). Stained cells were observed by fluorescence microscopy (Nikon, Chiyoda-Ku, Tokyo, Japan).

### Apoptosis

HK-2 cells were cultured in cell culture flasks (Eppendorf, Hamburg, Germany). Upon reaching 80% confluence, cells were exposed to different concentrations of Ox for 48 h. Cells were washed with cold PBS (4°C) and resuspended in 100 μl 1X Binding Buffer (BD Biosciences, San Jose, CA). Solution was transferred to a 1.5-mL tube; 10 μl PE Annexin V (BD Biosciences, San Jose, CA) and 10 μl P-AAD (BD Biosciences, San Jose, CA) was added to each tube. Samples were gently vortexed and incubated at room temperature for 30 min; 400 μl 1× binding buffer was added to each tube. Samples were analyzed by BD FACS-Calibur (BD Biosciences, San Jose, CA) within 1 h.

### Exosome isolation

Cell culture media (240 mL) were harvested from three groups (0, 1, and 2 mM) and centrifuged using a Beckman Coulter Allegra X-15R centrifuge (Beckman Coulter, Brea, CA) at 300×g (4°C) for 15 min, to remove detached cells. Supernatant was collected and centrifuged at 2000×g (4°C) for 15 min, to remove dead cells. Once again, supernatant was collected and centrifuged at 10000×g (4°C) for 30 min, to remove cellular debris. Then supernatant was collected and filtered through 0.45-mm filters (Merck Millipore, Billerica, MA) to remove apoptotic bodies and microvesicles. Clarified cell culture media were then centrifuged in a Beckman Coulter Optima L-XP Ultracentrifuge (Beckman Coulter, Brea, CA) at 110,000×g (4°C) for 90 min; swinging-bucket rotors were used to pellet exosomes. Supernatant was carefully removed, and crude exosome pellets were resuspended in 1.5 mL cold PBS (4°C). Crude exosome solution from each group was pooled in a single tube. Cold PBS was added to fill the tube completely prior to the second round of ultracentrifugation. Supernatant was carefully removed, and 100 μl cold PBS was added to each tube. Purified exosomes were gently resuspended, then stored at -80°C [19, 21, 22].

### Electron microscopy

A 20-μl drop of resuspended exosomes was placed on a sheet of Parafilm. Grids were transferred to the drops of exosomes for 3 min, then dried from the edge using filter paper (Solarbio, Beijing, China). The sample side of the membrane was then transferred to a 30-μl drop of 3% phosphotungstic acid solution, which was negative-stained at room temperature for 5 min. Then negative staining solution was removed from the grids with filter paper, and grids were dried at room temperature. Grids were observed with an H-7650 transmission electron microscope (Hitachi, Chiyoda-Ku, Tokyo, Japan).

### Nanoparticle tracking analysis

Each group of exosomes was diluted with cold PBS so that the concentration of particles ranged from 2 to 10×10^8^ particles/mL. NanoSight NTA LM10 (Malvern Instruments Ltd, Malvern, UK) was used to estimate the size distribution and number of particles in each group of exosomes.

### RNA extraction and quantification

Eight hundred microliters of TRIZOL (Invitrogen, Carlsbad, CA) was added to each sample of resuspended exosomes. Samples were gently mixed, then left to stand at room temperature for 5 min. Two hundred microliters of chloroform (Invitrogen, Carlsbad, CA) was added to each sample for two-dimensional separation. Ten micrograms of glycogen (without RNA enzyme, Invitrogen, Carlsbad, CA) were added before the addition of isopropanol (Invitrogen, Carlsbad, CA) to precipitate RNA. After two-dimensional separation, glycogen remained in the aqueous phase and co-precipitated with RNA during overnight storage at 4°C. NanoDropND-1000 (Thermo, Wilmington, DE) was used to estimate the concentration and purity of RNA.

### Protein extraction and quantification

Forty microliters of lysis buffer (RIPA:PMSF = 100:1, Solarbio, Beijing, China) was added to each sample of resuspended exosomes (50 μl/tube). Exosomes were lysed on ice for 30 min. Then protein concentrations were estimated with a BCA kit (Sigma, St. Louis, MO).

### Western blotting analysis

All proteins were resolved by 12% SDS-PAGE at 90 V for 90 min, then electrotransferred onto nitrocellulose membranes at 100 mA for 60 (CD9, Abcam, Cambridge, MA) or 100 min (CD63, SBI, Palo Alto, CA; HSP70, Abcam, Cambridge, MA). Nitrocellulose membranes were blocked with 5% (w/v) skim milk solution for 1 (CD63, HSP70) or 2 h (CD9), washed with TBST (Solarbio, Beijing, China) for 5 min, and incubated in primary antibody overnight on ice. Nitrocellulose membranes were washed with TBST for 10 min (three times) and incubated in secondary antibody at room temperature for 1 h. Membranes were then reacted with the ECL Substrate kit (ThermoFisher, Rockford, IL) and imaged with the ChemiDoc MP System (Bio-Rad, Santa Rosa, CA).

### Statistical analysis

Continuous variables are expressed as means ± standard deviations (SD). Comparisons between groups were performed using one-way ANOVA followed by LSD's post hoc test. *P* <0.05 and *P*<0.01 were considered statistically significant.
